# Forecasting the novel coronavirus COVID-19

**DOI:** 10.1371/journal.pone.0231236

**Published:** 2020-03-31

**Authors:** Fotios Petropoulos, Spyros Makridakis

**Affiliations:** 1 School of Management, University of Bath, Bath, United Kingdom; 2 Institute for the Future (IFF), University of Nicosia, Nicosia, Cyprus; Universidad Nacional de Mar del Plata, ARGENTINA

## Abstract

What will be the global impact of the novel coronavirus (COVID-19)? Answering this question requires accurate forecasting the spread of confirmed cases as well as analysis of the number of deaths and recoveries. Forecasting, however, requires ample historical data. At the same time, no prediction is certain as the future rarely repeats itself in the same way as the past. Moreover, forecasts are influenced by the reliability of the data, vested interests, and what variables are being predicted. Also, psychological factors play a significant role in how people perceive and react to the danger from the disease and the fear that it may affect them personally. This paper introduces an objective approach to predicting the continuation of the COVID-19 using a simple, but powerful method to do so. Assuming that the data used is reliable and that the future will continue to follow the past pattern of the disease, our forecasts suggest a continuing increase in the confirmed COVID-19 cases with sizable associated uncertainty. The risks are far from symmetric as underestimating its spread like a pandemic and not doing enough to contain it is much more severe than overspending and being over careful when it will not be needed. This paper describes the timeline of a live forecasting exercise with massive potential implications for planning and decision making and provides objective forecasts for the confirmed cases of COVID-19.

## 1 Introduction

The accuracy of traditional forecasting largely depends on the availability of data to base its predictions and estimates of uncertainty. In outbreaks of epidemics there is no data at all in the beginning and then limited as time passes, making predictions widely uncertain. On February 18, 2020, a New York Times article [1] cautioned against excessive optimism about the crisis peaking, even though there were close to 50 days since the virus had been identified.

Besides, there are concerns that the data may not be reliable, as was the case of bird flu and SARS when the number of affected people and deaths were misreported to hide the extent of the epidemic. Similarly, in the case of COVID-19, the reporting did not reflect the correct numbers as well when on the February 13 a new category of “clinically diagnosed” was added to “lab-confirmed” ones [2]. Such problems decrease forecasting accuracy and increase uncertainty, making the drawing of definite conclusions more difficult.

Related to forecasting accuracy and uncertainty, there is a more severe problem that has to do the perception of epidemics and pandemics. Politicians are concerned with regards to the measures to be taken while the general population fears about the impact on the epidemic on their health/lives. Furthermore, the pharmaceutical firms are working on vaccinations for the new virus with considerable commercial interest. This was the case with SARS when governments persuaded on the severity of the virus bought large numbers of vaccines that were never used as its spread stopped without the need to vaccinate people.

Of course, the big problem is the asymmetry of risks and the irrational fear of a pandemic with its possible catastrophic consequences, as happened with the 1918 Spanish flu that killed an estimated 50 million worldwide. In contrast, the SARS killed a total of 774 in 2003 and the bird flu around 100 in 1997. COVID-19 has resulted in an estimated 5.8 thousand deaths until now (15/03/2020). At the same time, there is much less concern over the seasonal flu that kills about 646,000 people worldwide each year [3].

Medical predictions are often not accurate while their uncertainty is seriously underestimated [4]. Predicting the future of epidemics and pandemics is much more difficult as the number of cases to be studied can be measured in one hand. At one end of the scale is the case of SARS where the fear of becoming a pandemic was overblown, resulting in overspending and the application of restrictive measures to be contained that it turned out to be unnecessary. At the other end is the Spanish flu that turned out to be a serious pandemic with catastrophic consequences, arguably in a different era when communication and the ability to raise public awareness (and possibly exaggerated fear) were limited.

Despite the inaccuracies associated with medical predictions, still forecasting is invaluable in allowing us to better understand the current situation and plan for the future. In this paper, we provide statistical forecasts for the confirmed cases of COVID-19 using robust time series models, and we analyse the trajectory of recovered cases.

## 2 Analysis and forecasting

**Fig 1:**
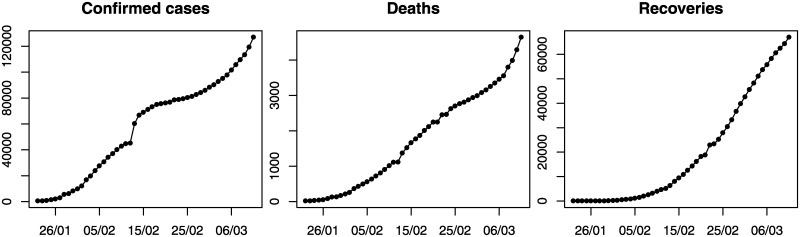
Daily cumulative confirmed, deaths and recovered cases from COVID-19.

We focus on the cumulative daily figures aggregated globally of the three main variables of interest: confirmed cases, deaths and recoveries. These were retrieved by the Center for Systems Science and Engineering (CSSE) at Johns Hopkins University (https://github.com/CSSEGISandData/COVID-19 accessed on 12/03/2020) and are presented in Fig 1. The data refer to daily cumulative cases and cover the period from January 22, 2020 until March 11, 2020. We include both “lab-confirmed” and “clinically diagnosed” cases. We emphasise the importance of the recovered cases, which is not covered in media as widely as the confirmed cases or the deaths. While all three data patterns show an exponential increase, the trends of both the confirmed cases and the deaths were reduced in the mid of February; a second exponential increase is observed in late February and March as a result of the increased number of cases in South Korea, Iran, and Europe. At the same time, the number of recovered cases is steadily increasing.

To forecast confirmed cases of COVID-19, we adopt simple time series forecasting approaches. We produce forecasts using models from the exponential smoothing family [5, 6]. This family has shown good forecast accuracy over several forecasting competitions [7–9] and is especially suitable for short series. Exponential smoothing models can capture a variety of trend and seasonal forecasting patterns (such as additive or multiplicative) and combinations of those. We limit our attention to trended and non-seasonal models, given the patterns observed in Fig 1. Note that we follow a pragmatic approach in that we assume that the trend will continue indefinitely in the future. This approach contradicts other modelling approaches to COVID-19 using an S-Curve model (logistics curve) that assumes convergence.

While statistical approaches to model selection (such as information criteria, which measure the maximum likelihood of a model while penalising for its complexity) could be used, we judgmentally select a model [10] to better reflect the nature of the data. We opt for an exponential smoothing model with multiplicative error and multiplicative trend components. Even if in some cases an additive trend model gave lower information criteria values, we opted for the multiplicative trend model given the asymmetric risks involved as we believe that it is better to err to the positive direction.

We produce ten-days-ahead point forecasts and prediction intervals and update our forecasts every ten days. Please note that this is not an ex-post analysis, but a real, live forecasting exercise. We have been posting and evaluating our forecasts publicly in social media (please, refer to the Twitter accounts of the authors, @fotpetr and @spyrosmakrid).

### First round of forecasts: 01/02/2020 till 10/02/2020

**Fig 2:**
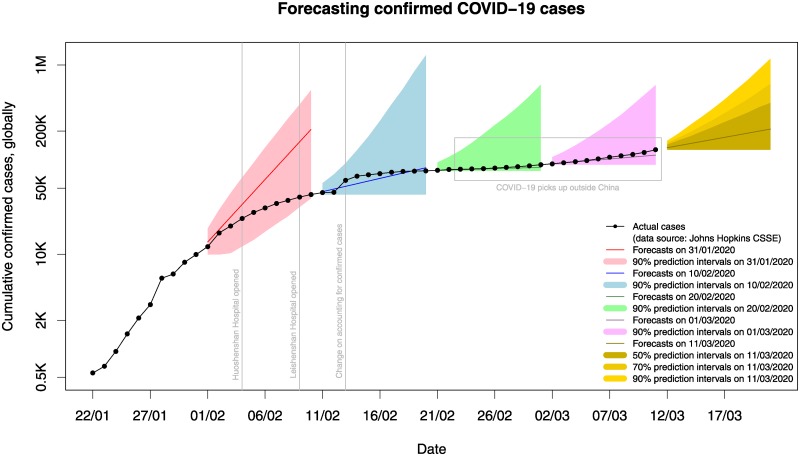
Cumulative actual confirmed cases of COVID-19, together with forecast and prediction intervals produced over several origins. The y-axis is log-scaled.

We first started at the end of January 31, 2020 and only had ten actual data points in hand. We decided to use a multiplicative trend exponential smoothing model. The forecasts (and 90% prediction intervals) produced at the end of 31/01/2020 are presented in Fig 2 with red (and pink) colour. Note that the vertical axis is log-scaled. The mean estimate (point forecast) for the confirmed cases ten-days-ahead was 209 thousand with the 90% prediction intervals ranging from about 38 to 534 thousand cases. The actual confirmed cases on 10/02/2020 were just under 43 thousand. We observe a considerable forecast error from the mean estimate equal to 166 thousand cases (an absolute percentage error of 388%), with the forecasts being extremely positively biased. Still, the actual cases lie within the prediction intervals.

### Second round of forecasts: 11/02/2020 till 20/02/2020

Then, we increased the historical number of our data to include cases up to the end of February 10, 2020 (20 data points). We once again produced ten-days-ahead predictions. The forecasts and prediction intervals are depicted in Fig 2 with blue colour. We observe that the actual values for the period 11/02/2020 until 20/02/2020 closely follow the mean estimate. The forecast error on 20/02/2020 was 5.8 thousand cases (an absolute percentage error of 7.7%). This was despite the change that was made on 13/02/2020 with regards to how confirmed cases are recorded to now include “clinically diagnosed” instances as opposed to exclusively lab-confirmed. One crucial observation is that this more accurate forecast came with a significant decrease in the steepness of the slope compared to the forecast for the previous ten-days period. Another observation is that at the end of 20/02/2020, we were still over-forecasting the number of confirmed cases. Finally, all actual values lied well inside the prediction intervals range.

### Third round of forecasts: 21/02/2020 till 01/03/2020

We produced a third set of forecasts and prediction intervals using the data up until 20/02/2020. The forecasts are presented in Fig 2 with green colour. The mean estimate for ten-days-ahead (01/03/2020) was 83 thousand cases. The slope of the forecasts was, once again, lower compared to the previous two sets of forecasts, confirming the fact that the observed confirmed cases (up until 20/02/2020) show a steady decrease. We also observed a significant decrease in the associated forecast uncertainty, with the prediction intervals being much tighter compared to our past forecasts. The 90% prediction intervals worst-case scenario was about 600 thousand cases, which is halved compared to that of the last round of forecasts (1.2 million cases). The actual confirmed cases at the end of 01/03/2020 were 88 thousand. At the end of this third round of forecasts, we recorded an error of 5.5 thousand cases (6.2%). While this error was lower than the previous round (in both absolute and percentage terms), it was the first time that our 10-days ahead forecast were below the actual values (under-forecasting). This was because the virus had been spreading in three countries outside Mainland China (South Korea, Iran, and Italy).

### Fourth round of forecasts: 02/03/2020 till 11/03/2020

Our fourth round of forecasts is shown in Fig 2 with purple colour. The mean estimate for 11/03/2020 was 112 thousand confirmed cases, with the uncertainty levels being similar to the previous round: There was a 5% chance that they would exceed 613 thousand by the end of 11/03/2020. The observed actual confirmed cases at the end of this period were almost 127 thousand. The absolute forecast error at the end of the last period (11/03/2020) was 15.4K (12.1%), higher compared to the previous set of forecasts but still well within the prediction intervals. For the second round in a row, we were consistently under-forecasting the actual cases. This was due to the exponential increase of the confirmed cases mostly in Europe, Iran and the US, with South Korea managing to decrease the number of new daily cases significantly.

### Fifth round of forecasts: 12/03/2020 till 21/03/2020

We produced a final set of forecasts and prediction intervals using the most recent data, up until 11/03/2020. These are presented in Fig 2 with yellow/gold colour. Note that we estimated three levels of uncertainty (50, 70 and 90%). The trend of our forecasts is much increased compared to the last two rounds: We predict 83 thousand new cases for this round (a total of 210 thousand cases). The associated levels of uncertainty are also increased: There is a 25% chance that the total confirmed cases will exceed 413 thousand by the end of 21/03/2020; and a 5% chance that they will exceed 1.19M.

We also attempted to produce forecasts by splitting the recorded confirmed cases into two groups: cases within Mainland China and cases anywhere else, as the trends into these two groups are different. We fitted independent exponential smoothing models, and then we summed up the forecasts (bottom-up hierarchical forecasting). We notice that using this approach, the mean estimate is close to that if all data are considered together (207 versus 210 thousand cases). However, the estimated uncertainty by splitting the data is considerably lower, possibly since the confirmed cases outside Mainland China have significantly increased only recently.

### Recovered cases

**Fig 3:**
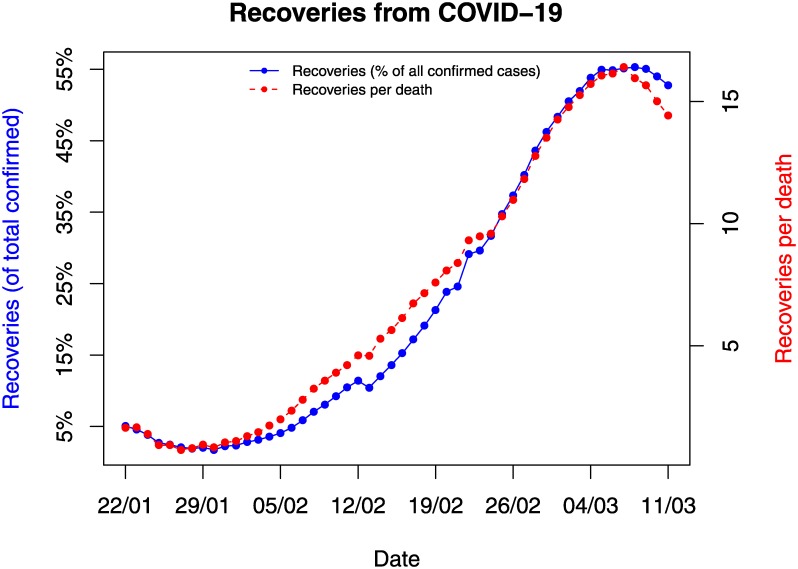
Recoveries as a percentage of the total confirmed cases and recovered cases per death over time.

We next turn our attention to the recovered cases that have received little attention until now. We focus on the number of the recovered cases as a percentage of the total confirmed cases as well as the ratio of recovered cases versus deaths. We are particularly interested in the trajectory of these two ratios. Fig 3 presents this analysis. First, we observe the solid relationship between the two curves. Second, we notice that despite the very small percentages of recovered cases until the end of January (less than 2%), currently, about 1 out of 2 confirmed cases have recovered (52.8% of the total confirmed cases). Moreover, the ratio of recovered cases versus deaths is currently above 14:1. Despite this, we observe a reverse of both curves since 08/03/2020, which is associated with the increasing number of cases outside Mainland China.

## 3 Discussion and conclusion

The uncertainty surrounding an unknown, novel coronavirus can spark a global alarm, leading a Harvard Professor stating that 40-70% of the global population might be infected in the coming year [11] which matches Chancellor Angela Merkel’s warning regarding the effects of the novel coronavirus in Germany [12]. Norman, Bar-Yam and Taleb [13] discuss the systemic risk of pandemics, the existence of fat-tailed processes due to global interconnectivity and the negatively biased estimates of spread, reproduction and mortality rates. On the opposite side, others are arguing about people overly panicking [14] and neglecting the probabilities [15, 16] with the new virus being the first “infodemic” as a result of the hyper-connectivity offered by today’s social media [17, 18]. The polarisation of the opinions globally can be summarised by the quotes of three renowned personalities:

- Elon Musk: “The coronavirus panic is dumb” [19].
- Nassim Nicholas Taleb: “Saying the coronavirus panic is dumb is dumb” [20].
- Bill Gates: “I hope it’s not that bad, but we should assume it will be until we know otherwise.” [21].

Regardless of what one’s beliefs are, we believe that forecasts and their associated uncertainty can and should be an integral part of the decision-making process, especially in high-risk cases. Apart from the significant public health concerns, the dangers imposed on global supply chains and the economy as a whole are also considerable [22, 23]. Risk-averse people can focus on the worst-case-scenarios and act accordingly. Deciding to discard any formal, statistical forecasts and acting conservatively, still implies an underlying forecasting process, even if this process is not formalised (personal judgment/belief).

In this exercise, we used univariate time series models, which assume that the data is accurate and past patterns (including precautionary measures) will continue to apply. Significant, consistent forecast errors (potentially spanning outside the prediction intervals) should be associated with changes in the observed patterns and the need for additional actions and measures in the case of negatively biased forecasts.

We believe that the significant forecast error at the end of the first forecast period (from 01/02/2020 to 10/20/2020) as depicted in Fig 2 could be the result of two factors:

- While the forecasts that we produced using the data up until 31/01/2020 would be a good estimate in the scenario of “business-as-usual” (nothing changes), they disregard the fact that the world *will* act to get the virus under control. The Chinese authorities managed to rapidly construct two new hospitals, in Huoshenshan and Leishenshan areas in Wuhan, that opened on 03/02/2020 and 08/02/2020 respectively. Multiple commuting restrictions were applied both within China and internationally. The World Health Organisation helped in creating awareness of the novel virus. So, the decline in the spread of the COVID-19 during this first round could well be linked with these attempts from local and global authorities.
- There may be a “garbage-in, garbage-out” situation. As mentioned above, our analysis and forecasts assumed that the data are accurate. It could be the case that the positive bias of the first-period forecasts is not as significant as it seems dues to potential inaccuracies in the actual data and the under-accounting of confirmed cases. This is especially true given the delay effects in diagnosing COVID-19 cases [24].

Our second and third sets of forecasts that cover the period 11/02/2020 to 01/03/2020 were very close to the recorded confirmed cases (the forecast error was lower than 6 thousand cases at the end of each 10-day period). The slowing down of the trend during this period suggested that COVID-19 would not cause any serious problems, particularly outside of Mainland China. Unfortunately, that was not the case. The last two sets of forecasts that cover the period 02/03/2020 to 21/03/2020 show a significant increase in the trend of cases globally coupled with an increase in the associated uncertainty. We hope that our forecasts will be a useful tool for governments and individuals towards making decisions and taking the appropriate actions to contain the spreading of the virus to the degree possible.
